# A rare presentation of a giant umbilical hernia in a patient with liver cirrhosis and chronic hepatitis B

**DOI:** 10.11604/pamj.2025.52.58.48074

**Published:** 2025-10-03

**Authors:** Mahima Dubey, Punam Sawarkar

**Affiliations:** 1 Department of Panchakarma, Mahatma Gandhi Ayurved College Hospital, Research Centre, Salod, Wardha, Datta Meghe Institute of Higher Education and Research, Maharashtra, India

**Keywords:** Hernia, umbilical cord, liver cirrhosis, hepatitis B, chronic

## Image in medicine

An individual aged 24 presents to the clinic with symptoms of abdominal bloating, decreased appetite, and sporadic abdominal pain. According to a general examination, the patient is afebrile, with stable vital signs, including a normal heart rate and blood pressure, despite being somewhat ill. Currently, the patient is on the following medications: 20 ml of Lactulose Syrup Nocte, 300 mg of tenofovir disoproxil fumarate tablet, OD, Lasilactone tablet (Furosemide 20 mg + Spironolactone 50 mg) BD. He has a notable medical history of chronic hepatitis B infection, portal hypertension, and liver cirrhosis. Additional signs of severe liver disease include palpable skin abnormalities close to the umbilicus and irreducible peripheral oedema. With a spot urine albumin of 3974.3 mg/L and creatinine of 198.97 mg/dL, recent laboratory tests revealed a very increased urine albumin-to-creatinine ratio of 1997 mg/g, indicating severe proteinuria. Computed tomography scan of abdomen and pelvis reveals gross free fluid in the abdomen and pelvis, along with the presence of mesenteric vascular congestion and haziness. Approximately one cm-sized defect is seen to be oozing into the hernial sac, which measures approximately 8.7x5.2 cm in size in the axial plane.

**Figure 1:**
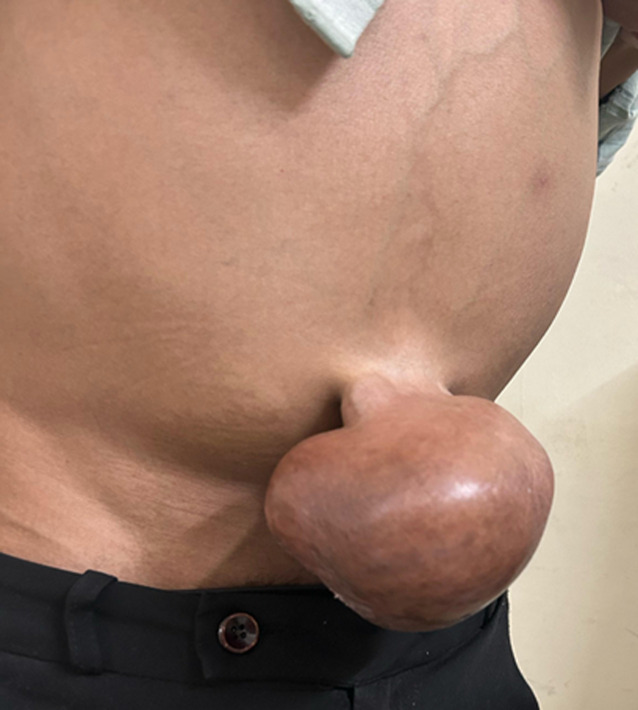
a prominent umbilical herniation observed in the orthostatic position

